# Detection of pathogenic *Escherichia coli* on potentially contaminated beef carcasses using cassette PCR and conventional PCR

**DOI:** 10.1186/s12866-019-1541-4

**Published:** 2019-07-30

**Authors:** Dammika P. Manage, Jana Lauzon, Christina M. Jones, Patrick J. Ward, Linda M. Pilarski, Patrick M. Pilarski, Lynn M. McMullen

**Affiliations:** 1 Department of Oncology, University of Alberta and Cross Cancer Institute, 11560 University Ave, Edmonton, AB T6G 1Z2 Canada; 2 Department of Agricultural, Food and Nutritional Science, University of Alberta, Edmonton, Alberta T6G 2P5 Canada; 3 Division of Physical Medicine & Rehabilitation, Department of Medicine, University of Alberta, 5-005 Katz Group Centre for Pharmacy and Health Research, Edmonton, AB T6G 2E1 Canada

**Keywords:** Cassette PCR, Validation study, Beef carcass swabs, STEC detection, O-antigen complexity

## Abstract

**Background:**

Over a one year period, swabs of 820 beef carcasses were tested for the presence of Shiga toxin-producing *Escherichia coli* by performing Polymerase Chain Reaction (PCR) in a novel technology termed “cassette PCR”, in comparison to conventional liquid PCR. Cassette PCR is inexpensive and ready-to-use. The operator need only add the sample and press “go”. Cassette PCR can simultaneously test multiple samples for multiple targets. Carcass swab samples were first tested for the presence of STEC genes (O157, *eae*, *stx1* and *stx2*). Samples were considered to be pathogenic if positive for *eae* plus *stx1* and/or *stx2*. For samples scored as pathogenic, further testing screened for 6 additional high frequency O-antigens (O26, O45, O103, O111, O121, and O145).

**Results:**

Of the 820 samples, 41% were pathogenic and 30% were O157 positive. Of these, 19% of samples were positive for O157 and carried potentially pathogenic *E. coli* (*eae* plus *stx1* and/or *stx2*). Of all samples identified as carrying pathogenic *E. coli*, 18.9, 38.8, 41.4, 0, 36.1, and 4.1% respectively were positive for O26, O45, O103, O111, O121, and O145. To validate cassette PCR testing, conventional PCR using STEC primers was performed on each of the 820 samples. Only 148 of 3280 cassette PCR tests were discordant with conventional PCR results. However, further fractional testing showed that 110 of these 148 PCRs reflected low numbers of *E. coli* in the enrichment broth and could be explained as due to Poisson limiting dilution of the template, affecting both cassette PCR and conventional PCR. Of the remaining 38 discordant tests, 27 initial capillary PCRs and 10 initial conventional tests were nominally discordant between cassette and conventional PCR, perhaps reflecting human/technical error on both sides of the comparison.

**Conclusions:**

Contaminated beef carcass swabs were often complex, likely harboring more than one strain of pathogenic *E. coli*. Cassette PCR had 98.8% concordance with parallel conventional PCR for detection of STEC genes. This indicates that cassette PCR is highly reliable for detecting multiple pathogens in beef carcass swabs from processing plants.

## Background

Although different strains of *Escherichia coli* are naturally occurring in the digestive tract of ruminant animals including cattle, shiga toxin-producing *E. coli* (STEC) are harmful to humans [1, 2]. Contamination of meat with STEC strains can occur at during evisceration and harvest of meat, making it one of the most common causes of food-borne diseases. Recalls of contaminated meat cost food industries millions of dollars. Meat testing is routinely conducted in abattoirs to detect potential contamination. Most testing strategies are time consuming with over 18 h or longer to detect 1–3 colony-forming units (cfu). Cassette PCR was developed to meet the need for simpler, cheaper and faster food testing [3] (Manage et al., in preparation).

The most commonly identified cause of infection with STEC strains is that of the O157:H7 serotype. However, the infections due to non-O157 STEC serotypes are on the rise [4–7]. North American food agencies target non-O157 STEC belonging to serogroups O26, O45, O103, O111, O121, and O145. These six serotypes are believed to be causing more than 70% of non-O157 STEC infections in the USA [8]. The main virulence factors of STEC strains include the production of Shiga-toxin (Stx) 1 or *2* and intimin (*eae*), an adhesion molecule that ensures toxin-producing *E. coli* remain firmly attached to the intestine (ehxA) localizing high toxin levels that cause hemolytic disease [6]. A strain of *E. coli* is considered to be pathogenic if it carries genes for *eae* as well as *stx1* and/or *stx2*. However, strains that carry only *stx2* are highly pathogenic [9], suggesting that other adhesion molecules can substitute for *eae*.

Current methods for detection of STEC strains involve culture-based methods, immunoassays, and DNA amplification methods, all of which require an enrichment procedure to increase the number of cells to a detectable level. Culture methods can be considered as the gold standard for detection of food-borne pathogens. However, their biggest drawback is the time required to obtain results, e.g. 24 to 48 h. The most commonly used DNA amplification method, Polymerase Chain Reaction (PCR), copies the target DNA, using specific primer sets. Conventional PCR is performed in a thermocycler in liquid media and the products can be detected in a variety of ways. In agarose gel electrophoresis, the amplicons are visualized by adding a dye to the gel. Capillary electrophoresis identifies product size using fluorescence to detect products. Amplification can be measured in situ by monitoring accumulation of fluorescent products in real time, sometimes in combination with Melt Curve Analysis (MCA) to verify product identity; this is the method used for cassette PCR. MCA is based on measuring the DNA denaturation point (T_m_) that confirms product identity, based mainly on GC content and size of the amplicon.

Cassette PCR successfully detects pathogens and single nucleotide polymorphisms in raw body fluids or in food products [10–14]. PCR is performed in semi-solid gel, followed by in situ MCA in a capillary reaction unit (~ 6 μL volume) that contains all reagents, including a primer set and Taq polymerase, with arrays of capillaries assembled in each cassette. Each capillary contains only one primer set to detect one target. An array of capillaries, each with a different primer set, allows simultaneous detection of multiple targets in parallel. Cassettes can be assembled to detect the desired panel of targets. The sample is administered to the cassette via capillary forces with no pumps or applied pressure involved. The cassette geometry can be altered for the test requirement. These pre-made cassettes can be stored for at least 7 months at 4 °C or − 20 °C [10] and up to 3 years at 4 °C (unpublished results). With this technology, we have previously detected multiple sexually transmitted diseases for multiple patients with raw urine and genital swabs [11] and single nucleotide polymorphisms in breast cancer genes tested using buccal swabs [12]. With a somewhat different architecture for making reaction gels, we also amplified BK virus in whole blood with no purification involved [13].

Over a one year period, 820 beef carcasses from provincially inspected processing plants in Alberta were swabbed to evaluate contamination with potentially pathogenic *E. coli* using two detection methods, cassette PCR and liquid tube-based PCR, here termed conventional PCR. Swabs were collected several times per week over a year, from January to December of 2016 from several small provincial abattoirs in Alberta, and processed by Alberta Agriculture and Forestry (AAF). An aliquot of each enriched sample was tested by cassette and conventional PCR. Cassette PCR was validated to be 98.8% concordant with conventional PCR. Analysis of STEC targets as well as O-antigen genotypes indicated that carcass swab samples are highly complex, containing multiple strains of pathogenic *E. coli*. Pathogenicity, defined here as presence of *eae* plus *stx1* and/or *stx2*, was detected in 41% of swab samples. Either or both of *stx1* and *stx2* was found in 58% of swab samples, with all O-antigens represented except O111.

## Results

### Cassette PCR

**Fig. 1:**
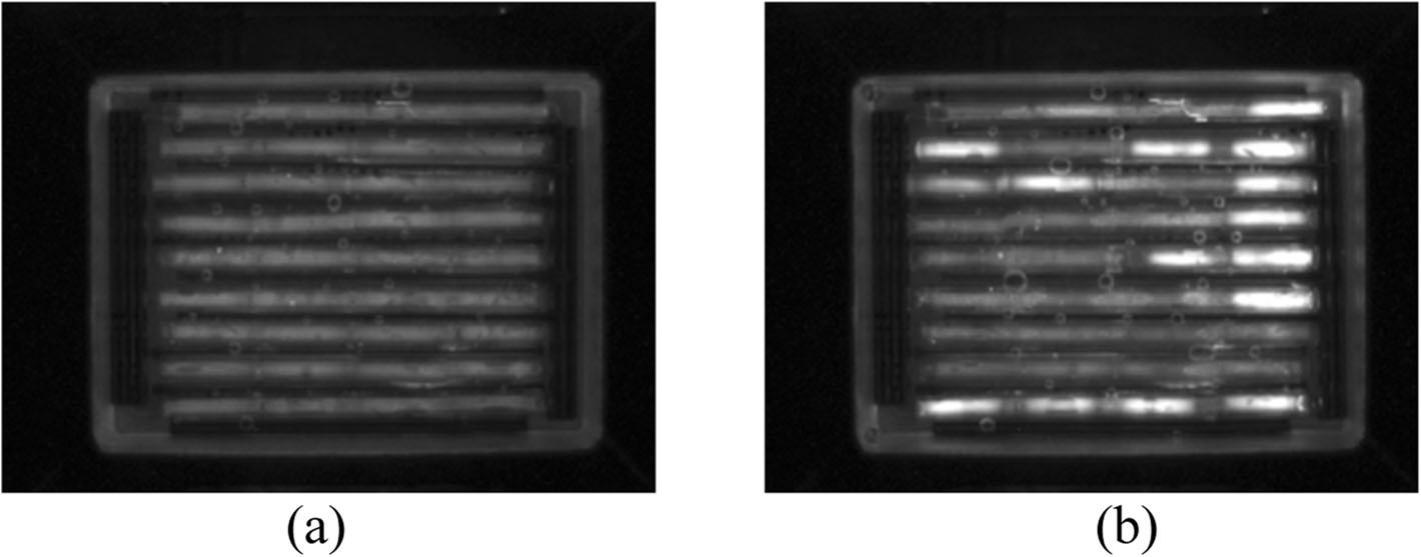
CCD images of a cassette with 9 trenches and 4 sets of reaction units per trench (**a**) at 1st PCR cycle and (**b**) at 35th cycle. Four columns from left to right in 9 trenches (rows) have O157, eae, stx1 and stx2 primers. The last trench (9th) has the positive control and 8th trench has the negative control. Trenches 1–7 have 7 carcass swab samples

PCR was performed in the cassette containing 36 capillaries in 9 trenches where seven samples were tested in the first 7 trenches and negative and positive controls were tested in the last two trenches respectively for four STEC primer sets. Images taken after cycle 1 and cycle 35 of the PCR are shown in Fig. 1 for a STEC cassette.

**Fig. 2:**
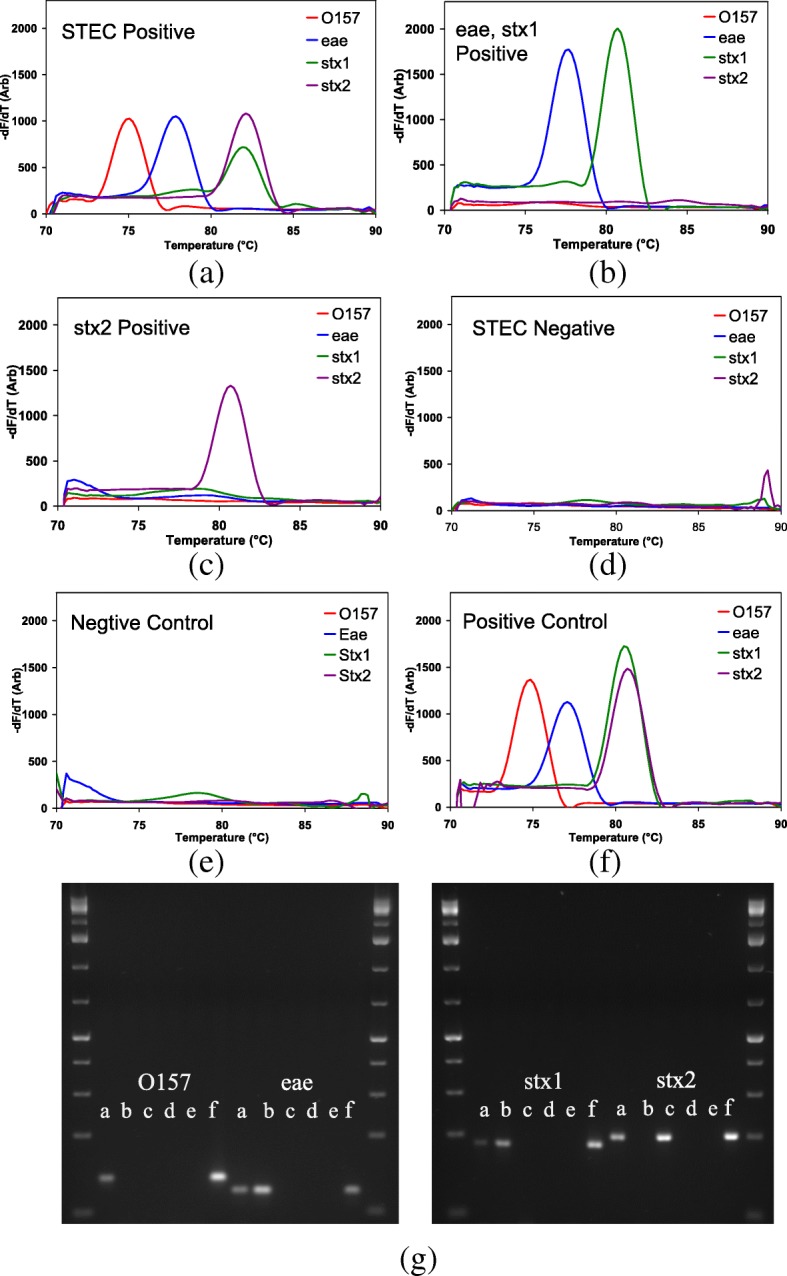
Melt curve analysis data of the cassette for: four carcass swab samples; (**a**) a STEC positive; (**b**) positive for *eae* and *stx1* only; (**c**) positive for *stx2* only; (**d**) a STEC negative; and for (**e**) the negative control (water); (**f**) the positive control of the cassette; and (**g**) shows agarose gel images with the conventional PCR products of the same samples used in (**a**), (**b**), (**c**), and (**d**), as well as the controls used in (**e**) and (**f**). The ladder is GeneRuler 1kb Plus DNA ladder (Themo Scientific, Carlsbad, USA)

Melt peaks of four different samples are shown in Fig. 2 representing some of the various patterns of STEC results observed in this study. Patterns of positivity vary from all four PCR reactions scoring positive to all four PCR reactions scoring negative.

The gel image in Fig. 2(g) shows the PCR products from the conventional PCRs run with four STEC primers corresponding to the samples in (a)-(f).

### STEC pathogen contamination in beef carcass swabs

For 820 carcass swab samples, we analyzed cassette PCR results using all four primer sets (O157, *eae*, *stx1*, *stx2*). Analysis of cassette PCR results for *stx1*, *stx2* and *eae* categorized 338 samples as “pathogenic” (*eae* and *stx1* and/or *stx2* positive), as shown in Table 1. As indicated below, the results from cassette PCR testing as shown in the Tables were concordant with parallel testing by conventional PCR.

**Table 1:** The number and percentage of enrichment broths from carcass swab samples obtained from January to December, 2016 that were positive for STEC by cassette PCR

		Number and percent (in brackets) of samples scoring positive			
Month	total # samples	*eae* plus *stx1* and/or *stx2*	*stx1*	*stx2*	Both *stx1* and *stx2*
January	42	18 (42.9)	9 (21.4)	25 (59.5)	9 (21.4)
February	42	18 (42.9)	15 (35.7)	24 (57.1)	11 (26.2)
March	64	26 (40.6)	16 (25.0)	35 (54.7)	13 (20.3)
April	88	23 (26.1)	20 (22.7)	38 (43.2)	13 (14.8)
May	56	28 (50.0)	20 (35.7)	32 (57.1)	16 (28.6)
June	52	36 (69.2)	17 (32.7)	30 (57.7)	8 (15.4)
July	56	42 (75.0)	32 (57.1)	39 (69.6)	26 (46.4)
August	44	16 (36.4)	7 (15.9)	19 (43.2)	7 (15.9)
September	72	37 (51.4)	24 (33.3)	44 (61.1)	22 (30.6)
October	62	21 (33.9)	11 (17.7)	33 (53.2)	10 (16.1)
November	134	49 (36.6)	43 (32.1)	74 (55.2)	37 (27.6)
December	108	24 (22.2)	23 (21.3)	38 (35.2)	16 (14.8)
Total	820	338 (41.2)	237 (28.9)	431 (52.6)	188 (22.9)

**Fig. 3:**
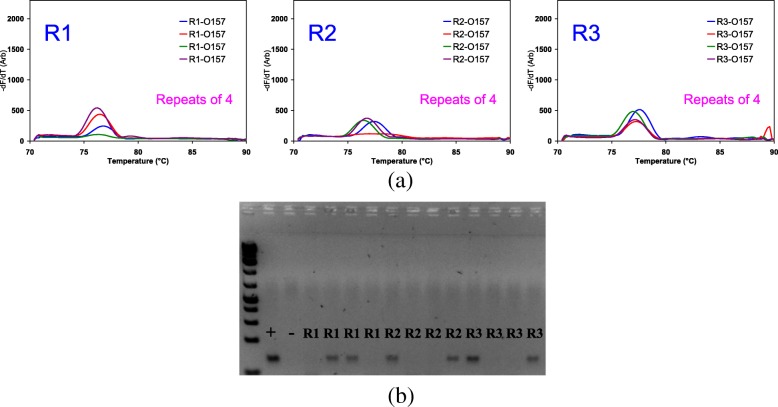
Quadruple PCRs performed in gel capillaries and in conventional PCR on three carcass swab samples. **a** Melt peaks of R1, R2 and R3 and (**b**) agarose gel image showing the ladder and the bands for PCR products. The ladder is GeneRuler 1 kb Plus DNA ladder (Themo Scientific)

For the 820 samples, 3280 individual PCRs were performed in cassette PCR and also in conventional PCR, with four STEC primers. In cassette PCR, 3132 reactions matched with conventional PCR. Only 148 reactions appeared to be non-concordant between the two methods. Some of the 148 out of 3280 reactions that did not match those for conventional PCR, or vice versa, gave weak peaks or bands, suggesting that template might be limiting and that a fractional analysis was required. These 148 PCRs were replicated 4–8 times in both cassette PCR and with conventional PCR in tubes (examples shown in Fig. 3).

About half of the 820 samples were positive for *stx2* (53%) and slightly over a quarter of samples carried *stx1* (29%) (Table 1). Of these, 70 and 79% of samples with *stx2* and *stx1,* respectively*,* carried *eae*, defining them as having pathogenic *E. coli*. A total of 30 and 21% of samples that were positive for *stx2* and *stx1*, respectively, lacked *eae* (Table 2), presumptively preventing them from adhering to the intestinal cells and thereby localizing toxin production.

**Table 2:** Number and percentage (in brackets) of enrichment broths from carcass swab samples that were positive for *stx1* and/or *stx2* but lacked *eae*. Data is from cassette PCR results

Month	total # samples	*stx1* + ve	*stx1* + ve but *eae* –ve	*stx2* + ve	*stx2* + ve but *eae* –ve	*stx1*&*2* + ve	*stx1*&*2* + ve but *eae* -ve
January	42	9	3 (33.3)	25	7 (28.0)	9	3 (33.3)
February	42	15	3 (20.0)	24	8 (33.3)	11	1 (9.1)
March	64	16	3 (18.8)	35	12 (34.3)	13	3 (23.1)
April	88	20	10 (50.0)	38	18 (47.4)	13	6 (46.2)
May	56	20	1 (5.0)	32	8 (25.0)	16	1 (6.3)
June	52	17	1 (5.9)	30	2 (6.7)	8	0 (0)
July	56	32	0 (0)	39	3 (7.7)	26	0 (0)
August	44	7	1 (14.3)	19	3 (15.8)	7	1 (14.3)
September	72	24	3 (12.5)	44	9 (20.5)	22	3 (13.6)
October	62	11	4 (36.4)	33	13 (39.4)	10	4 (40.0)
November	134	43	11 (25.6)	74	28 (37.8)	37	8 (21.6)
December	108	23	9 (39.1)	38	19 (50.0)	16	7 (43.8)
Total	820	237	49 (20.7)	431	130 (30.2)	188	37 (19.7)

For the 338 samples with a pathogenic genotype, a total of 2366 PCRs were performed using seven primer sets to detect the seven most frequent O-antigens (Table 3).

**Table 3:** O-antigen genotypes in samples scoring positive for pathogenic *E. coli*, from January to December 2016. Data is from cassette PCR results

		Number and percent (in brackets) of pathogen-positive samples						
Month	total # pathogen+ samples	O26	O45	O103	O111	O121	O145	O157
January	18	1 (5.6)	5 (27.8)	11(61.1)	0 (0)	0 (0)	0 (0)	3 (16.7)
February	18	1 (5.6)	5 (27.8)	8 (44.4)	0 (0)	0 (0)	1 (5.6)	4 (22.2)
March	26	2 (7.7)	6 (23.1)	5 (19.2)	0 (0)	6 (23.1)	0 (0)	6 (23.1)
April	23	1 (4.3)	6 (26.1)	4 (17.4)	0 (0)	6 (26.1)	0 (0)	6 (26.1)
May	28	1 (3.6)	19 (67.9)	13 (46.4)	0 (0)	13 (46.4)	2 (7.1)	11 (39.3)
June	36	11 (30.6)	19 (52.8)	22 (61.1)	0 (0)	14 (38.9)	2 (5.6)	16 (44.4)
July	42	23 (54.8)	19 (45.2)	29 (69.0)	0 (0)	28 (66.7)	1 (2.4)	27 (64.3)
August	16	6 (37.5)	9 (56.3)	12 (75.0)	0 (0)	13 (81.3)	0 (0)	13 (81.3)
September	37	7 (18.9)	16 (43.2)	16 (43.2)	0 (0)	13 (35.1)	1 (2.7)	28 (75.7)
October	21	2 (9.5)	10 (47.6)	4 (19.0)	0 (0)	5 (23.8)	0 (0)	12 (57.1)
November	49	5 (10.2)	11 (22.4)	6 (12.2)	0 (0)	18 (36.7)	1 (2.0)	24 (49.9)
December	24	4 (16.7)	6 (25.0)	10 (41.7)	0 (0)	6 (25.0)	6 (25.0)	8 (33.3)
Total	338	64 (18.9)	131 (38.8)	140 (41.4)	0 (0)	122 (36.1)	14 (4.1)	158 (46.7)

As shown in Table 4, in the month of January, we performed the full panel of O-antigen testing for all the samples irrespective of their pathogenicity. Enrichment broths from carcass swabs contained a complex mixture of strains of *E. coli*, including those lacking the seven most frequent O-antigens.

**Table 4:** Results for the presence of O-antigens in enrichment broths received in the month of January

	# of samples negative for O-antigens	# of samples positive for O-antigens						
		O26	O45	O103	O111	O121	O145	O157
Non-pathogenic samples (*n* = 24)	13	0	4	6	0	1	0	0
Pathogen+ samples (*n* = 18)	3	1	5	11	0	0	0	3

To evaluate complexity of the *E. coli* strains in any given enrichment broth, we determined the number of O-antigens that we present in the table. The presence or absence of genes for seven different O-antigens were tested by PCR on the pathogenic samples. For any given sample, the number of detectable O-antigens ranged from zero to 6 (O111 being absent from all samples tested). The number of O-antigen genotypes present in each of the 338 pathogen–positive carcass swab samples are shown in Table 5.

**Table 5:** Complex mixtures of *E. coli* strains with more than one O-antigen strain per sample

	# of different O-antigens in a given sample							
	1	2	3	4	5	6	7	None
# of pathogenic samples	100	87	54	34	9	2	0	52
% in pathogenic samples (*n* = 338)	29	26	16	10	3	2	0	15
% of O157+ pathogenic samples (*n* = 158)	20	27	27	20	6	1	0	0

## Discussion

In summary, this analysis of carcass swabs demonstrated that they can carry complex mixtures of pathogenic *E. coli*. Individual swabs often harbored more than one strain of pathogenic *E. coli*. Analysis using a new testing technology, cassette PCR, indicated that cassette PCR had almost complete concordance with parallel conventional PCR for detection of STEC genes, indicating that cassette PCR is highly reliable for detecting multiple pathogens in beef carcass swabs from processing plants.

Cassette PCR was monitored via the increase in fluorescence of the LC Green dye, an intercalator that fluoresces only if bound to double stranded DNA [15]. As the PCR progresses by producing more and more double strand amplicons, the fluorescence increases. The fluorescent images of the cassette are taken by CCD camera during the PCR and MCA imaging all the capillaries at once.

As shown in the cassette image in Fig. 1(b), upon completion of PCR, there is an increase in fluorescence in some of the capillaries due to the double stranded DNA intercalated with LCGreen dye. When the temperature is raised during the MCA, double stranded DNA separates to single strands at the melting temperature of the PCR product causing the dye to disassociate, resulting in a sharp drop in the fluorescence. Plotting the first derivative of the fluorescence signal with the temperature produces a peak revealing the melt temperature of the product. Since this melting temperature is dependent on the GC content, length and the sequence of the product, it is possible to use it as a marker to determine the PCR amplification of the correct target.

Therefore, the presence or the absence of a melt peak at the correct temperature in a given capillary with a given primer set determines the presence or absence of the target DNA template in any given sample. The temperature of the melt peak for a given primer was defined by the peak position for the positive control with the same primer set, with a tolerance of ±1 °C. Peak intensity was determined as the extent to which a test peak rises above the intensity of the same primer in the negative control (water). Therefore, the positive peak is defined as a peak being at the correct temperature with an intensity above the background level. If such a peak is present in the MCA analysis, the PCR in the corresponding capillary is considered to be “positive”.

### Concordance between the cassette PCR and conventional PCR

Comparison of the MCA results from 3280 individual capillaries with individual conventional PCRs revealed that only 148 reactions are non-concordant. As shown in Fig. 3, templates of some of these 148 samples might be limited. Fractional analysis revealed that 110 samples had results expected for a Poisson limiting dilution pattern where the template copy number is limiting. This indicates that cassette and conventional PCR were in fact concordant for these samples, both showing limiting dilution effects. For a further 27 out of 3280 cassette PCR tests and 10 conventional PCR tests, the initial results could not be repeated. These tests most probably reflect human or technical error. One sample repeated the original data even with replicates. However, if we arbitrarily classify these as non-concordant samples, this shows that for cassette PCR, 98.8% of tests were concordant between cassette and conventional PCR for the same samples. Thus, for cassette PCR, 1.2% of reactions appear to be nominally non-concordant in presumed process errors that affected conventional PCR as well as cassette PCR.

Even after nearly 20 h of enrichment, 110 PCRs had limiting copy numbers. Figure 3 shows quadruple PCRs run with the O157 primer set for three samples where cassette and conventional PCR gave different results that were resolved using fractional analysis. It should be noted that the amount of sample added in both to the capillary and to the conventional PCR is approximately the same (~ 6 μL per reaction unit or tube). R1, R2, and R3 samples were positive for cassette PCR 3/4, 3/4, 4/4 replicates for each sample, respectively, while 2/4, 2/4, and 2/4 were positive for conventional PCR, respectively, both showing the effects of a limiting dilution.

The swab samples used here were biologically complex samples with multiple species including microflora. During the enrichment, these multiple strains/species of *E. coli* and other bacteria would compete for nutrients for growth. Valadez *et. al.* showed that the presence of multiple strains with different O-antigens added together in low numbers caused some not to grow [16]. When cultures were enriched individually with similar numbers, they were able to grow. In the current study, the swabs and enrichment broths may contain varying amounts of blood and other biological matter that could be inhibitory to the PCR. Competition among the microbiota and the presence of inhibitors may explain the weaker PCRs even after the samples were enriched for a considerable time.

Over the year long study of carcass swabs, we found that during the summer months nearly all samples carrying *stx1* and/or *stx2* also carried *eae*, defining them as harboring pathogenic *E. coli*. Though these are the two primary types of Shiga toxin, the *stx2* gene has been identified as a high risk factor for bloody diarrhea and haemolytic uremic syndrome [9, 17], even when *eae* is absent, perhaps reflecting the contributions of adhesion molecules other than intimin. Co-expression of *stx1* and *stx2* may result in reduced toxicity compared to expression solely of *stx2*, although differences in serotype or host background are also likely to influence pathogenicity [18]. However, in carcass swab samples it is not possible to confirm that *stx1* and *stx2* are carried by the same cell for cases where both scored positive in testing.

### More frequent O-antigens detected in carcass swab samples

For the seven O-antigens included in our screening, all were detected except O111. All primer sets were verified as accurate in cassette PCR by testing with known strains of *E. coli*. Other than for O157, O antigen status was not tested using conventional PCR. Testing for O157 by cassette and conventional PCR was concordant.

In contrast to the six non-O157 O-antigens that were tested only among enrichment broths shown to carry pathogenic *E. coli*, all 820 broths were tested for presence of O157, as part of the STEC panel of O157, eae, stx1 and stx2 primer sets. In 820 samples, 245 enrichment broths had O157, and 158 (64.5%) of these also carry pathogenic *E. coli*. This indicated that O157 is present even in the apparent absence of *eae* and *stx* genes. However, among those O157 positive samples that lacked *eae*, 25/87 (29%) did score positive for *stx2*, a genotype known to cause severe symptoms even when *eae* is absent [9].

Among the total of 42 samples tested for a full panel of O-antigens (for the month of January), only 36% had a detectable O-antigen as well as the markers for pathogenicity (*eae* and *stx*). However, 26% carried a detectable O-antigen even though they lacked a detectable STEC genotype. Thus, O-antigen positivity cannot be considered by itself to denote pathogenicity. Never the less, among the apparent pathogen-negative but O-antigen-positive samples, 5/11 (45%) did harbor *stx2*.

Overall, of the 338 pathogen-positive samples tested, when the number of O-antigens in a given sample increases to > 1, that sample tended to also carry O157. Of 338 enrichment broths scoring positive for *eae* and *stx1* and/or *stx2* (termed pathogenic) tested for O-antigen genotypes, 186 (55%) carried two or more O-antigens, demonstrating the potential for the presence of more than one strain of pathogenic *E. coli* (Table 5). This highlighted the extensive complexity of *E. coli* populations in processing plant swabs. Significantly, 15% of pathogenic swab samples lacked any of the 7 major O-antigen strains, suggesting that they carried rare O-antigens. Of the pathogen-positive samples, 9% carried only O157, again confirming the complexity of the swabs and the observation that O157 tends to be present together with other O-antigen strains of *E. coli*. The majority of samples that are O157-negative also lack any of the other major O-antigens, indicating the pathogenic *E. coli* present in these samples must carry unknown O-antigen genotypes.

Foodborne outbreaks caused by non-O157 serotypes in the world, up to 2004 and in the USA up to 2010, were summarized by Bettelheim [5] and Luna-Gierke *et. al.* respectively [19]. Luna-Gierke *et. al.* reported 46 outbreaks in the USA, with 1727 illnesses and 144 hospitalizations. For outbreaks where the pathogen could be confirmed, over half involved STEC O111 or O26. The rest included small numbers of O45, O103, O121, O145, O104, O165, and O undetermined. Although O111 was the most common antigen in American outbreaks until 2010, surprisingly, we did not detect any O111 among our 820 samples. In Ontario, Bannon et al. found that O103, O45 and O121 were the most common strains identified on carcasses and the plant environment, but all six serotypes were represented [20]. The deadly 2011 outbreak in Germany was attributed to non-O157 strains, specifically to O104:H4 [18]. Considering the number of illnesses reported with non-O157 infections, together with our data showing the presence of carcass-localized strains lacking any of the most frequent O-antigens (Table 5), raises the issue of whether or not O-antigen testing has value for food safety screening. A significant number of pathogenic *E. coli* may be missed if the focus is primarily on O-antigen screening.

This analysis of samples collected over a full year shows that there was an increase in pathogen levels during the summer. This has been observed previously when detecting STEC in cattle feces samples collected during Jan-Mar and June-Aug from 24 pen floors [21]: O45, O111, and O121 were not detected during the summer months. In contrast, we detected more O-antigens during the summer months for all detected O-antigens, including O157, except for O145. Venegas-Vargas *et. al* studied the shedding of STEC pathogens in 1,096 cattle from six dairy and five beef herds in the summers of 2011 and 2012. They found that the STEC pathogen shedding frequencies vary considerably across cattle herds in Michigan and shedding of non-O157 serotypes far exceeds shedding frequencies of O157, which is congruent with human infections in the state.

## Conclusions

Cassette PCR used in this study for screening 820 carcass swab samples had 98.8% concordance with parallel conventional PCR testing of the same samples. We show that about 41% of beef carcass swabs carry pathogenic markers of *eae* plus *stx1* and/or *stx2*. Carcass swab samples harbor complex mixtures of *E. coli* strains with as many as six different O-antigens detectable in a single swab sample. In addition, a substantial proportion of swab samples lacked any of the most frequent O-antigens, indicating that testing solely for O-antigens, which do not play a major role in pathogenicity, is likely to miss some pathogenic strains. Testing for *ea*e and *stx* genes, the mediators of pathogenicity, is essential for maintaining food safety. Cassette PCR, a simple, inexpensive and automated platform to screen food for its safety, contains capillary reaction units holding all the reagents needed for PCR except the sample. Each cassette can test multiple samples for multiple pathogens, including positive and negative controls for each cassette. Since the sample is delivered via capillary forces with no complex methods involved, relatively unskilled personnel can load the cassette and run the PCR while still ensuring accurate results. With this large number of samples and even larger number of individual tests, our validation data set showing 98.8% concordance with the conventional standard PCR tests confirms the reliability of the ready-to-use cassette PCR for detecting pathogens in food.

## Materials and methods

### Samples

Carcass sponge swabs were collected from provincially-licensed abattoirs in Alberta during January – December 2016 by AAF. Sponge samples were enriched at 42 °C for 15-24 h in modified Tryptic Soy Broth (mTSB, OXOID, Basingstoke, England). Aliquots of the enriched media were provided for testing by cassette and conventional PCR. A total of 82 batches of enriched samples were received from AAF; the number of samples varied from 2 to 26 samples per batch. With each batch of samples, a sterility control (enriched uninoculated media), a positive control (enriched *E. coli* O157:H7 ATCC 35150), and a negative control (enriched media with *E. coli* ATCC 25922) were provided by AAF. For processing, 8 μL of the enriched sample was added to 72 μL of a proprietary buffer (Amplicet Inc., Edmonton AB, Canada). This mix was heated at 55 °C for 15 min and then to 97 °C for 4 min. The freshly processed sample was then used to rehydrate the capillary reaction units: 25 μl was delivered per trench containing 4 capillary reaction units.

### Cassette PCR

The sequences of the primers used to detect STEC and O-antigens are shown in Table 6. Separate reaction mixes were prepared with each of these primer sets to fill separate capillaries. Each 100 μL reaction mix consisted of 20 μL of 5xPCR buffer [333 mmol/L tris sulfate, pH 8.6, 83 mmol/L (NH4)_2_SO_4_ (Sigma, St. Louis, USA) and 40% sucrose (Sigma)], 30 μL of 40% trehalose (Cargill Inc., Winnipeg, Canada), 4 μL of 50 mmol/L MgCl_2_ (Fluka, Buchs, Switzerland), 2 μL of 10 mmol/L dNTP (Sigma), 2 μL of 1% bovine serum albumin (Sigma), 4 μL of 10 μM primer solution for O157, stx1 and stx2, and 6 μL of 10 μM primer solution for eae (Integrated DNA Technologies, San Diego, USA), 10 μL of 10x LC Green Plus (Idaho Technology Inc., Salt Lake City, USA), 4 μL of 20 U/mL Taq polymerase, 10 μL of a 40% acrylamide (Sigma) + 4% bis-acrylamide aqueous solution (N,N-methylene bisacrylamide; Bio-Rad, Hercules, USA), 2 μL of 3% azobis (Wako Bioproducts, Richmond, USA), 1 μL of 10% N,N,N′,N′ tetramethylethylenediamine (Sigma), and water. The mixes were vortexed, centrifuged, and loaded into the capillaries.

**Table 6:** Primer sequences for STEC and O-antigen amplifications

Primer Name	Sequence	Length (bp)	Reference
O157-F	TCG TGA CAA CCA TTC CAC CTT	123	This work
O157-R	GCG CTG AAG CCT TTG GTT CT		
eae-F	CAT TGA TCA GGA TTT TTC TGG TGA TA	102	[22, 23]
eae-R	CTC ATG CGG AAA TAG CCG TTA		
stx1-F	GTG GCA AGA GCG ATG TTA CGG TTT G	182	[24]
stx1-R	ATG ATA GTC AGG CAG GAC GCT ACT C		
stx2-F	ACG AGG GCT TGA TGT CTA TCA GGC G	200	[24]
stx2-R	GCG ACA CGT TGC AGA GTG GTA TAA C		
O26-F	GTA TCG CTG AAA TTA GAA GCG C	158	[23, 25]
O26-R	AGT TGA AAC ACC CGT AAT GGC		
O45-F	TAT GAC AGG CAC ATG GAT CTG TGG	132	[26]
O45-R	CAC AAC GCA ACG AAA GTC CC		[27]
O103-F	AAT TGC TCT ATG CGC TCT TCC	136	This work
O103-R	GCC CAC CAT AGA TAA CAA CGA		
O111-F	GGA ATA ATC GAC CGG CCA AA	199	This work
O111-R	TAA TGT GTT GCC TCG CCT TC		
O121-F	TGT TGG CTA GTG GCA TTC TGA	212	This work
O121-R	TTC TGC ATC ACC AGT CCA GA		[26]
O145-F	AAA CTG GGA TTG GAC GTG G	135	[25]
O145-R	CCC AAA ACT TCT AGG CCC G		

### Cassette preparation

**Fig. 4:**
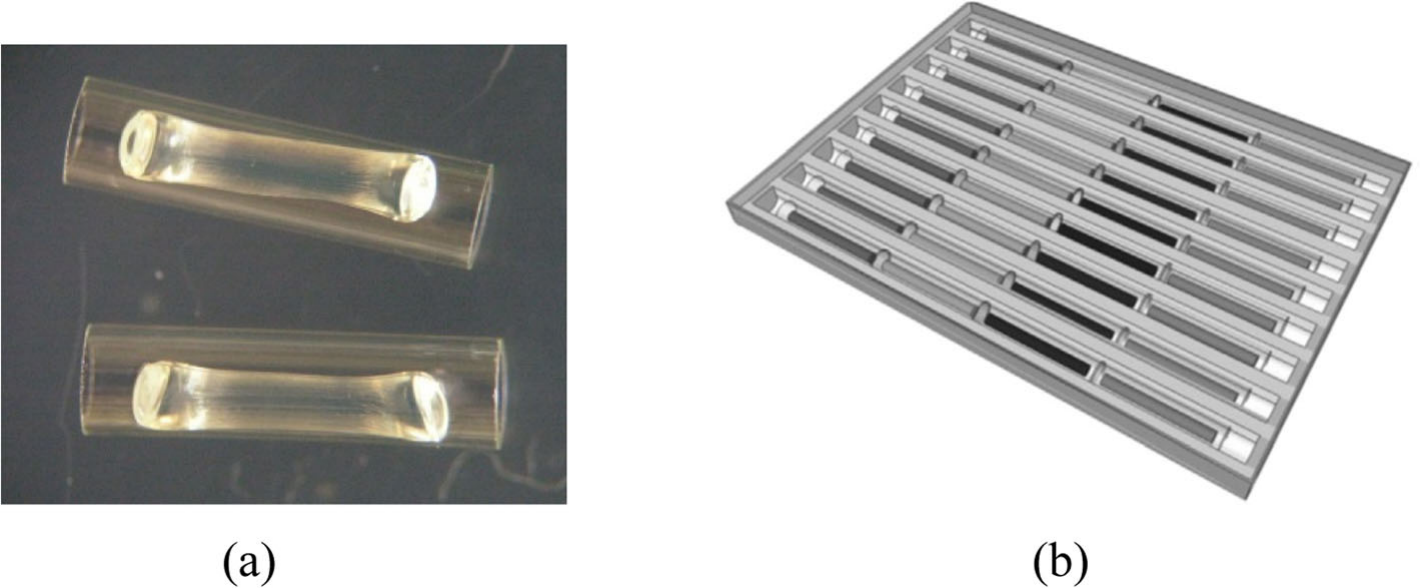
Reaction capillaries and an assembled cassette for cassette PCR (**a**) Photograph of capillaries with dried gel inside. (**b**) Pan with a 4 × 9 capillary array ready-to-use cassette for STEC testing

The method for constructing capillaries was published previously in detail [14]. Briefly, glass capillaries (with 1.1 mm inner diameter) were custom cut to 6 mm pieces, heated to 550 °C overnight to remove any pre-coating, filled with reaction/gel mix, polymerized for 30 min under 370 nm wavelength, and desiccated overnight at a pressure of 81.27 Pa. After desiccation, capillaries hold dried gel in the shape of a “noodle” (Fig. 4a). For delivering the sample to the capillary, the noodle shape of the dried gel is vital as the space created between the gel noodle and the glass capillary walls creates a path for the sample to flow by capillary force and thereby rehydrate the gel.

For laying capillaries, trench imprints were made in an aluminum pan (23.5 mm × 32 mm) filled with 1 mL of wax (Surgipath Paraplast X-tra; Leica Microsystems, Deerfield, USA) with a polydimethylsiloxane stamp by heating wax to its melting temperature of 56 °C, as previously described [11, 14]. The shape of the bottom of the trench has a curvature similar to that of the capillary, such that the capillary fits snugly in the wax trench. The capillaries with desiccated gels were placed in each trench of a pan with nine trenches. An example of a STEC cassette is shown in Fig. 4b. It contains 9 trenches where 7 trenches are used to test 7 samples while the last two trenches are used as integrated negative and positive controls for each cassette. Once the wax is melted, the liquid wax covers the capillaries and acts as a vapor barrier. For testing the six O-antigens, three capillaries (O26, O45, and O103) were placed in one trench while the other three capillaries (O111, O121, and O145) were placed in the second trench. Nine trench cassettes can test 2 samples for O-antigens with 4 trenches reserved for positive and negative controls. When testing many samples for O-antigens, two separate pans were made, each testing three antigens per trench. This allows testing for O-antigens of up to 7 samples with controls in two pans.

### Sample delivery

The processed sample (25 μL) was administered into the 1st capillary of each trench holding four capillaries (in the STEC pan) and 19 μL each to the two trenches holding three capillaries in the O-antigen cassette, in order to hydrate the gels with the sample. Negative control capillaries were hydrated with water. For the positive control capillaries, a known positive control sample from AAF was used. Desiccated gels inside the capillaries need about 10 min to hydrate.

For conventional PCR tests, 6 μL of the enriched carcass swab sample was added to each reaction. For the positive and negative control PCRs, a positive control from AAF and water were used respectively. The latter confirms the sterility of the cassette.

### PCR and MCA

PCR was performed on cassettes with hydrated gel capillaries in a prototype instrument explained earlier [11]. Briefly, it contains a Peltier device for heating and cooling, a laser for fluorescence excitation, and a CCD camera to acquire the images during PCR and MCA. They are controlled by a microprocessor. A laptop computer running a customized Java-based program was used to control the instrument. The DNA amplification was performed with a pre-denaturation step of 94 °C for 3 min, then 35 cycles of 94 °C for 15 s, 59 °C for 20 s, and 72 °C for 20 s, followed by a final amplification of 72 °C for 2 min. Upon the completion of the PCR, MCA was performed by heating the cassette from 70 °C to 90 °C and the CCD images were taken at 0.2 °C degree intervals. They were analyzed to measure the melting temperature for amplicons in each capillary with ImageJ software (National Institutes of Health, U.S.) using the MicroArray Rectangular Plug-in (Dr Robert Dougherty, OptiNav Inc., Redmond, WA) to plot the negative derivative of the fluorescence with respect to the temperature in order to determine the melting temperature (T_m_) of the PCR products.

Initially the samples were tested for the presence of the four STEC genes (O157, *eae, stx1* and *stx2*). If the sample was pathogenic, PCR was performed with a panel of O-antigen primer sets.

### Conventional PCR

For comparison of the data obtained using cassette PCR, conventional PCR was performed on all 820 samples. Separate reaction mixes were prepared with each of the O157, *eae*, *stx1*, and *stx2* primer sets (Table 6). Each 25 μL reaction mix consisted of 5 μL of 5xPCR buffer, 0.5 μL of 50 mmol/L MgCl_2_, 0.5 μL of 10 mmol/L dNTP, 0.3 μL of 2% bovine serum albumin, 0.5 μL of 10 μM primer solution for stx1 and stx2, and 1.5 μL of 10 μM primer solution for O157 and eae, 0.1 μL of 20 U/mL Taq polymerase, 6 μL of sample and water. Thermal cycling was performed with a pre-denaturation step of 94 °C for 3 min, then 35 cycles of 94 °C for 20 s, 58 °C for 30 s, and 72 °C for 30 s, followed by a final amplification of 72 °C for 2 min in a thermocycler (Applied Biosystems, Foster City, USA). PCR products were visualized in 2% agarose gels containing SYBR Safe DNA gel stain (Invitrogen, Carlsbad, USA). Only the STEC primers were tested using conventional PCR (4 tests per sample for 820 samples).

## Data Availability

All the data required is included in the manuscript.

## Abbreviations

AAF: Alberta Agriculture and Forestry
CCD: Charged Coupled Device
DNA: Deoxyribonucleic acid
*E. coli*: *Escherichia coli*
MCA: Melt Curve Analysis
PCR: Polymerase Chain Reaction
STEC: Shiga toxin-producing *E. coli*
